# Removing constraints to sustainable food production: new ways to exploit secondary metabolism from companion planting and GM

**DOI:** 10.1002/ps.5508

**Published:** 2019-07-09

**Authors:** John A Pickett, Charles AO Midega, Jimmy Pittchar, Zeyaur R Khan

**Affiliations:** ^1^ School of Chemistry University of Cardiff Cardiff United Kingdom; ^2^ Push‐pull IPM Technology, Plant Health, International Centre of Insect Physiology and Ecology Nairobi Kenya

**Keywords:** companion planting, agro‐ecological manipulation, GM, genetic modification, transgenic technologies, plant secondary metabolism

## Abstract

The entire process of agricultural and horticultural food production is unsustainable as practiced by current highly intensive industrial systems. Energy consumption is particularly intensive for cultivation, and for fertilizer production and its incorporation into soil. Provision of nitrogen contributes a major source of the greenhouse gas, N_2_O. All losses due to pests, diseases and weeds are of food for which the carbon footprint has already been committed and so crop protection becomes an even greater concern. The rapidly increasing global need for food and the aggravation of associated problems by the effects of climate change create a need for new and sustainable crop protection. The overall requirement for sustainability is to remove seasonal inputs, and consequently all crop protection will need to be delivered via the seed or other planting material. Although genetic modification (GM) has transformed the prospects of sustainable crop protection, considerably more development is essential for the realisation of the full potential of GM and thereby consumer acceptability. Secondary plant metabolism offers wider and perhaps more robust new crop protection via GM and can be accomplished without associated yield loss because of the low level of photosynthate diverted for plant defence by secondary metabolism. Toxic mechanisms can continue to be targeted but exploiting non‐toxic regulatory and signalling mechanisms should be the ultimate objective. There are many problems facing these proposals, both technical and social, and these are discussed but it is certainly not possible to stay where we are in terms of sustainability. The evidence for success is mounting and the technical opportunities from secondary plant metabolism are discussed here. © 2019 The Authors. *Pest Management Science* published by John Wiley & Sons Ltd on behalf of Society of Chemical Industry.

## 1 INTRODUCTION

In 2009 a peer‐reviewed report was published by the Royal Society (UK) on the findings of a working group, chaired by Sir David Baulcombe FRS, on the science for sustainable intensification of global agriculture on a 10 year timescale.^1^ A well‐evidenced case was presented for this need and, although many related pleas have been made since,^2^, ^3^ this pioneering review of the literature‐based evidence stands as a powerful commendation for action. The actions included a major redirection of, and increase in, research into sustainable systems for food production and also substantially raised resourcing for the food production infrastructure, not least the training of agronomists for the new demands of industrialising sustainable agricultural technologies. The 10 years are almost up and more money is indeed being spent on research into food sustainability worldwide but it is insufficient to fulfil the main recommendations of the 2009 report. The main funding worldwide is still being spent on non‐sustainable approaches, particularly those employing seasonally purchased inputs relating to seed, crop nutrition and pest control.^1^, ^4^ The 2009 report explains that the technologies needed for the future are very different from those currently based on the highly successful technologies for intensification of agricultural production of the post Second World War green revolution. There are major research and development targets dealing with both plant nutrition and protection, and all require delivery via the seed and other planting material for sustainability. Undoubtedly, use of genetic modification (GM), and all new and newly emerging technologies under this general classification, is required. In parts of the world, particularly mainland Europe and many sub‐Saharan African countries, this is a problem of public perception, with many agencies fuelling unevidenced concern among potential consumers for human environmental safety. The risks from GM being applied to solving the problems of lack of sustainability in agricultural food production are extremely low, but the perceived hazard can be high as a consequence of attempts to regulate, as currently with genome editing tools, the technology rather than the products obtained using these GM technologies. The fulfilment of demands for sustainable agricultural technologies requires great innovation and the new tools needed, which are predicted in the 2009 report, are only very slowly emerging through a lack of resources and prioritisation. Therefore, food production will continue to rely on the conventional technologies of fertilizers and pesticides, which are themselves greatly in need of defence against baseless criticism.^3^ It is already likely, because of unevidenced concerns regarding non‐target effects of insecticides and herbicides, that there will be far fewer commercially developed pesticides registered in the future for use in Europe.

Breeding clearly contributes to sustainability but is restricted to providing traits too close to the current main crop species and requires seasonal application, particularly for hybrids. Thus, there must be a major effort to replace the current arable cropping systems with perennial plants that will remove the seasonal high‐energy land preparation^1^ but will raise further problems which will also need early attention in this process. Without access to genetic material beyond the crop species there will be insufficient diversity to solve the sustainability problems with conventional breeding.

The major success of insect‐resistant transgenic crops expressing genes encoding *Bacillus thuringiensis* endotoxins (Bt crops) required access to genes even from another living kingdom and the plethora of conventional breeding programmes currently funded worldwide for weed and invertebrate pest management is not likely to succeed without the capture of much more taxonomic diversity of protein based resistance for use by GM.

There are many inherent problems for even GM providing the new traits for sustainable nitrogen fixation and the essential improvements in the wider efficiency of nutrient and water usage, as well as photosynthesis.^3^ In addition it is proposed^1^ that these traits will need to be combined with the requirement for new improved agronomic traits such as perennialisation. To meet these challenges there is an urgent need for policy makers to rise above debating the approaches and accept what new technologies from GM that we have while advancing risk assessment to cover the specific risks of the new GM products. The demand for crop protection may appear less daunting but this does not accommodate the likely prospect that the toxic protein‐based insect resistance of Bt, exploited currently, may be less widespread in nature. So far, alternative toxicants in this class have been slow to emerge. In addition, new GM‐based insect resistance traits will need to be extremely powerful because, until sustainability of crop production is fully achieved, the carbon footprint of all food lost to pests, diseases and weed competition is already committed. Thus, substantially higher levels of crop protection efficacy will be required to justify the continued carbon footprint of non‐sustainable practices.

The relatively little movement towards the goals of the 2009 report on sustainable intensification of global agriculture^1^ strongly suggests that the continued aggravating circumstance of climate change and increases in global human population have not been sufficiently taken into account. This sadly may not happen until there are food shortages in the industrialised countries. Food prices continue to rise and already there is an associated weakening of infrastructural support for the poorest of these communities. This has contributed to changes in global political priorities, causing rising health problems related to access to nutritionally appropriate food. In developing countries the situation is substantially worse, although often hidden by the rise of a more economically privileged middle class, with vast numbers, including small‐holder farmers, not only denied nutritionally valuable food but often lacking food calories. Industrialised countries often appear to justify their aid budgets by the need to help provide food security in order to offset northward migration and the radicalisation of communities, thereby aggravating terrorism.

Perhaps the most insidious argument for not doing enough to bring about the second and greener revolution in agriculture is that there is a belief that the problem will disappear. This has happened in the past, particularly in connection with food production. One change that can be expected without immediate intervention is that the inevitable rise in food prices, brought about by climate change and increased world population, will shift economic power from the food processing and retailing industries to production, and the farmers and industries that support their needs in terms of currently seasonal inputs. This could help the move to sustainable technologies, including from perennial seed and other planting material, to more impacting decision support technologies. There will be other gains because we are no longer served well by a food industry already operating a monopoly approach to maintain profitability. This is also an industry with a very low research activity noted more for taking commercial advantage from the adulteration of primary food commodities not only with water but also salt, sugar and still currently unsustainably produced fats and with a delivery approach based on waste and inbuilt costs offering further mark up. This rise in the cost of food against income could not only allow but even promote more sustainable food production by funding and even encouraging new science, particularly GM, and the building of a more prestigiously regarded work force providing sufficient and healthier food for a world changing from largely entertainment‐based high‐carbon footprint gadgets to an interest in healthier living. Thus in the setting of these issues relating to the essential intensification of sustainable food production, the technical opportunities, particularly from exploiting secondary plant metabolism, are presented.

## 2 CROP PROTECTION FROM SECONDARY PLANT METABOLISM BY COMPANION PLANTING

Agents active as selective toxicants such as natural insecticides can be delivered by companion plants, but the approach is not as efficient as separate production, and application loses the sustainable value of natural production. However, relatively recently the concept of plant volatile‐mediated signalling and its application to agriculture has started to receive considerable attention. Delivery by agro‐ecological management techniques such as companion planting, and particularly the push‐pull or stimulo‐deterrent diversionary strategy system involving companion intercrops that repel pests from the crop plants (push) and plants growing around the crop plants to capture the pests (pull), and where some method of population reduction can be achieved are now showing excellent results.^5^ Thus, the companion plants are selected, from plant species diversity, naturally to produce the signals or semiochemicals that effect the appropriate repellency or attraction. Companion planting is difficult to imagine becoming mainstream for industrialised agriculture, but it can be feasible for small‐holder farming in developing countries where the farmers have no access to seasonally purchased inputs with which to deal with constraints such as pest attack. Lessons learnt from successful companion planting can also be valuable for building new GM approaches in which the crop plants themselves produce and even respond to the plant‐derived semiochemicals.^6^ Related signalling in the rhizosphere shows promise for crop protection^7^ and relates to allelopathic effects which can include direct physiological mechanisms as well as signalling.^8^

### 2.1 Plant‐derived semiochemicals via companion planting

**Figure 1:**
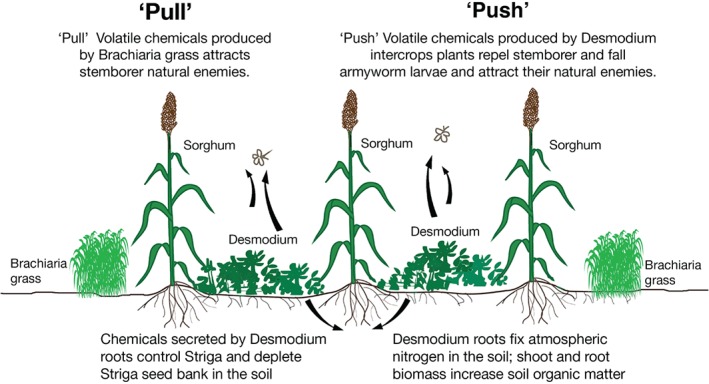
The push‐pull for the control of stemborer moth larval damage to cereals on small holder farms in sub‐Saharan Africa is explained in this annotated cartoon, and in the text,^5^, ^9^ for the further control of the parasitic weed Striga hermonthica^8^, ^10^ and more recently for the fall armyworm.^11^, ^12^ This push‐pull intervention against the pests and the agro‐ecological advantages of plant nutrition and other factors^13^ gives three fold cereal yields with no seasonal inputs of fertilizer or pesticide even under climate change stress.^14^, ^15^ The intervention comprises grass and legume perennial companion crops, highly valuable as farm animal forage bringing further value to the small holder farms. The chemistry underpinning these processes is defined and offers possibilities for exploitation by genetic modification of the food crops themselves which work at the field scale providing clear evidence that manipulation of pests and weeds by these plant secondary metabolites can be usefully effective^6^.

The most advanced system of companion planting for delivering plant‐derived semiochemicals is the push‐pull system for removing insect and parasitic weed constraints in small‐holder farming in sub‐Saharan African cereal production (Fig. 1). This push‐pull system was initially developed for control of stem borer moth pests, including indigenous Noctuidae and introduced Crambidae that oviposit in the cereals, followed by larval penetration of the stems causing lodging and massive yield losses. Companion plants that act as the push were discovered empirically initially and subsequently, by chemical analysis and gas chromatography coupled electrophysiology using the insect antennae, found to repel moths from sorghum and maize cereal crops by releasing the oxidative stress‐related isoprenoids (*E*)‐4,8‐dimethyl‐1,3,7‐nonatriene (DMNT) and (*E,E*)‐4,8,12‐trimethyl‐1,3,7,11‐tridecatetraene (TMTT). Particularly for release of DMNT, the perennial cattle forage molasses grass *Melinis minutiflora* was identified for both repulsion (push) of gravid female pests, including *Busseola fusca*, Noctuidae, and *Chilo Partellus*, Crambidae, and for increasing foraging of larval parasitoids, e.g. *Cotesia sesamiae* and Braconidae, and used highly successfully on the farm.^9^ Initially, the trap crops (pull) which were grown, also perennially, surrounding the combined cereal and the intercrop were, by farmer's choice from a number of suitable forage grasses producing high levels of attractancy, mainly Napier grass, *Pennisetum purpureum*.^5^, ^13^ Further farmers' concerns introduced a survey of legumes as alternatives to molasses grass that would additionally fix nitrogen and led to the discovery of the perennial forage legume silverleaf, *Desmodium uncinatum*, Fabaceae. This intercrop not only fulfilled the push role against the stem borers but was extremely powerful, by an allelopathic mechanism, in controlling the other major constraint on small‐holder cereal farming, the parasitic witchweed *Striga hermonthica*, Orobanchaceae.^5^, ^13^

### 2.2 Climate‐smart push‐pull for cereal farming in sub‐Saharan Africa

With the very high impact of anthropogenic climate change on low‐input small‐holder farming in sub‐Saharan Africa, and with EU development funding (EU‐ADOPT followed by EU‐IBCARP) climate smart and particularly drought tolerant push‐pull was developed^5^, ^13^ by the initial replacement of the main crop of maize with the intrinsically more drought‐tolerant sorghum. Showing greater tolerance to drought the *D. uncinatum* was replaced with *D. intortum* with a more drought‐tolerant trap crop provided by an apomictic cultivar of a savannah forage grass genus, *Brachiaria* cv Mulato II. In degraded soil and with high pressure from pests and parasitic weeds, dramatic increase in cereal yields,^14^, ^15^ for example of sorghum from under 0.5 t ha^−1^ (equivalent) to nearly 3 t ha^−1^, could be obtained on farms with no other inputs than the perennially growing companion crops. This is set to improve further by the introduction of even more drought‐tolerant and insect‐resistant *Brachiaria* spp. cultivars and newly identified intercrops such as *D. incanum*.^10^

### 2.3 Other constraints, including fall armyworm, managed by the small‐holder cereals push‐pull

In addition to controlling pest and weed constraints, the value of livestock forage has been invaluable to small‐holder farmers taking up this push‐pull approach,^13^ and other advantages beyond the obvious provision of fixed nitrogen include a reduction in aflatoxin levels in maize from push‐pull farms when compared with mono‐maize cultivation. In addition, the push‐pull system sequesters higher levels of carbon and thereby contributes to mitigation of the intrinsic carbon footprint from industrial agriculture. Perhaps the most dramatic recent development of push‐pull is the demonstration in Kenya, Tanzania and Uganda of almost complete avoidance of attack by the fall armyworm, *Spodoptera frugiperda*, Noctuidae, by push‐pull farms compared with normal farming practice.^11^, ^12^ Over 250 000 small‐holder farms are now practicing this type of push‐pull and enjoy maize yields over three times normal farmer practice and without the need for seasonal inputs.^12^

## 3 CROP PROTECTION FROM SECONDARY PLANT METABOLISM BY METABOLIC ENGINEERING

The need for new science for the sustainable intensification of global agriculture by current and advancing GM technologies has been introduced. In addition to there being more opportunities than from pesticidal proteins, e.g. Bt crops against insect pests, the secondary metabolites of plants already show a growing opportunity as, or for the development of, insecticides.^16^ By choosing highly biologically active compounds from the wide range of natural products available, including insect toxicants, pheromones and other semiochemicals as well as some allelopathic agents, there will be little loss of photosynthate via this form of defence and with the latest GM technologies no other detracting phenotypic changes, for example Bruce *et al*.^17^

The problem of low acceptance and application of potential GM of crops includes aspects such as (i) particularly in Europe, the opposition to evidence‐based recommendations for registration of GM of crops by political groups opposed to the use of GM crops on the grounds of the precautionary principle, (ii) lack of public awareness of the scientific principles and benefits of the transgene technology, particularly in less developed countries, (iii) lack of a legal framework to commercialize GM of crops and where this is approved, as in Kenya, a still existing moratorium against environmental release and trade of GM of foods, particularly maize, and (iv) in most African countries small‐holder farmers rarely have the financial means to annually purchase expensive seed. These views and issues will be moderated by the social and political effects of food shortages but more particularly require evidence that GM can realise its potential in providing sustainable intensification of food production. However, underpinning these measures, wider engagement with the public on current concerns regarding GM is essential.

### 3.1 Insecticides via GM and beyond

Biosynthetic pathways to plant secondary metabolites with exploitable insecticidal activity,^16^ by definition, exist in nature and so can already be considered as potential targets for development by GM. The obvious example for this approach to exploiting natural products with insecticidal activity is the lead compound, pyrethrin 1 from *Tanecetum cinariifolium* (Asteraceae), for the synthetic pyrethroid insecticides. This is under investigation^18^ and involves bringing together as the ester an unusual monoterpenoid structure for the chrysanthemic acid component and the oxidative metabolic product, pyrethrolone, of the unsaturated fatty acid, linolenic acid, as the alcohol component.^19^ This has not yet been achieved (Matsuda, personal communication). More recently, the betenolide insecticides, e.g. flupyradifurone, have been developed to mimic the lead insecticidal natural product stemofoline from *Stemona japonica* (Stemonaceae).^16^ The isobutenolide head group of the natural product has been essentially maintained in flupyradifurone but the complicated cage structure comprising an isoprenoidal alkaloid replaced by a much simpler group incorporating a 2‐chloropyrid‐5‐yl structure.^20^ The biosynthesis of stemofoline by *S. japonica* has been elucidated,^21^ which could provide a route to stemofoline in crop plants by GM but a rational synthetic biology approach could be adopted as has been successful for other plant derived isoprenoids^22^ and which can also deliver halogen substitution by means of halogenated precursors in the isoprenoid pathway. Alternatively, halogen incorporation can be effected by enzymes from marine algae potentially facilitated by industrial production, for example of the edible red alga*, Porphyra umbilicalis* (Bangiaceae).^23^ Thus not only natural product insecticides, or natural product lead compounds for synthetic insecticides, could be exploited by GM approaches but by use of rapidly developing synthetic biology the biosynthesis of non‐natural insecticides can now be targeted for exploitation by GM in a new generation of crops with control of pest insects as robust as that achieved by current synthetic insecticides comprising small molecule lipophilic toxicants. This approach should lend itself also to the other major crop protection agents, including fungicides and herbicides, all of which either relate directly to natural products^16^ or can now be envisioned as being biosynthesised in crop plants by enzyme systems developed via the concepts of synthetic biology. Furthermore, because biosynthesis based on GM could, in addition to protecting crop plants directly, be transferred to rapidly sexually cycling microbes for their protection against other microbes, an approach based on natural evolution to addressing pesticide resistance could be investigated.^24^

### 3.2 Pest pheromones delivered by GM

In the early 1980s pest pheromone delivery by GM was proposed^25^ but GM technologies were not sufficiently developed until later and then only in the model plant *Arabidopsis thaliana*, Brassicaceae.^26^ The expression of genes for the synthesis of the aphid alarm pheromone comprising (*E*)‐*beta*‐farnesene conferred an alarm response involving repellency to aphids, *Myzus persicae*, Aphididae, and increased foraging for aphid prey by the parasitoid wasp *Diaeretiella rapae*, Braconidae, which kills aphids feeding on members of the Brassicaceae. The elite wheat cultivar Cadenza was then stably transformed using synthetic gene constructs, with appropriate codon usage, giving constitutive expression and transit peptides for localization within the plastids of the (*E*)‐*beta*‐farnesene synthase with and without a synthase for farnesyl diphosphate, the pheromone biosynthetic precursor, originally from a bovine sequence.^17^ Excellent results were obtained in the laboratory against cereal aphid species and with cereal aphid parasitoids, but in replicated field studies with two spring sowings and one autumn no aphid control or increase in parasitism was observed.^17^ Appropriate levels for activity of (*E*)‐*beta*‐farnesene, at approximately ten‐fold higher for the line with the enhanced farnesyl diphosphate, were obtained in the field but the constitutive release profile is different from the sudden burst of pheromone naturally produced. Habituation, although known to occur, was unlikely to have occurred in these field conditions.^17^The two lines of Cadenza, other than the production of (*E*)‐*beta*‐farnesene, showed no phenotypic differences, clearly demonstrating that modification of this plant's secondary metabolism to produce the insect pheromonal component had no demonstrable metabolic cost and certainly not in terms of crop stature, seed yield or chlorophyll content.^17^ Thus, to imitate precisely the mode by which the alarm pheromone is produced naturally is the next step in exploiting this new opportunity for crop protection.

Alarm pheromones and other pheromones transmitting negative messages are the most obvious GM targets, but pheromones associated with other types of behaviour including the sex pheromones, already being investigated by GM for biotechnological production, can be considered. The aphid sex pheromones, comprising specific isomers of the iridoids (cyclopentanoids), nepetalactone and nepetalactol,^27^, ^28^ are strategically targeted for development by GM because, although these attract only autumn flying male aphids, they are powerful foraging cues for aphid parasitoids at this vulnerable time in the host alternating aphid life cycle. Towards this objective, new reports on the aphid sex pheromone biosynthesis and associated molecular genetics in aphids, rather than on similar compounds produced by plants, are in preparation (Partridge, SJ, *et a*l. in preparation)

### 3.3 Plant defence‐related semiochemicals delivered by GM

Obviously, there are problems associated with the exploitation of pheromones by GM relating to the physical release from plant tissue rather than from specialised organs in animals. However, while this can be potentially solved using aphid attack to promote synthetic gene function in plants, an alternative is to target defence‐related semiochemicals that are produced when plants are attacked and which deter further attack. Examples of such compounds have been introduced and include the products of isoprenoid oxidation, DMNT and TMTT, see Section 2.1. These work well when delivered by companion planting, particularly against cereal pests and in small‐holder farming where other less sustainable approaches involving seasonal purchase of insecticides is not economically viable. For industrial agriculture, such highly volatile and chemically unstable compounds cannot be formulated in a cost‐effective manner, so use of GM to generate crops that can more effectively use these semiochemicals is appealing. Already the crop plant rice has been genetically modified to produce increased levels of DMNT and TMTT, and in laboratory studies increased the foraging behaviour of the parasitoid, *C. chilonis*, Braconidae, attacking the rice pest *Chilo suppressalis*, Crambidae.^29^ This study is a prelude to attempting control of the brown planthopper*, Nilaparvata lugens,* Delphacidae, the major rice pest.

### 3.4 Allelopathic control of weeds delivered by GM

Because of the very close association with their hosts, parasitic weeds are extremely difficult to control selectively.^8^ However, the control of witchweeds in the *Striga* genus by intercropping with *Desmodium* ssp., although difficult to explain in evolutionary terms, is highly selective and with no detectable damage to the cereal hosts including maize, sorghum, the millets and rain‐fed rice as introduced earlier. The associated allelopathic agents are di‐*C*‐glycosylated flavones,^30^ and the biosynthesis pathway and some of the molecular genetics have been elucidated, raising the prospect of an additional GM route to exploit this chemistry in addition to the already totally sustainable intercropping approach. Seed availability has been problematic, but is being solved as evidenced by current farmer take‐up.^12^

## 4 INDUCTION AND PRIMING OF SECONDARY METABOLITE DEFENCE

An important aspect of natural plant defence is that although secondary metabolism diverts only a small amount of photosynthate there has been evolutionary pressure against natural selection for resistance by widespread employment in the plant kingdom of induction and priming. The plants utilised in companion plant systems such as push‐pull and even the crops can demonstrate this. However, for most GM developments for pest control to date, including current Bt crops, expression of insect controlling genes is constitutive. Thus natural plant defence and in particular push‐pull incorporate signals for defence priming and induction, thereby offering the signals themselves as defence elicitors or activators and the associated promoter sequences for the initiation of induction and priming.^6^

### 4.1 Induction and priming with plant volatile‐mediated signalling

Plants produce the volatile methyl esters of the disease and pest damage‐related hormones salicylic and jasmonic acids. Salicylic acid activity has been explored extensively in industry and synthetic analogues created, e.g. acibenzolar‐S‐methyl, and developed commercially. For jasmonic acid, this type of activity has largely been abandoned by industry because of associated phytotoxic effects. However, many plant‐derived volatile compounds released during pest attack can induce some form of defence without directly having potentially damaging hormonal roles. Of these, *cis*‐Jasmone has been widely studied, including in the field, for both inducible and also priming effects.^6^, ^31^, ^32^ Cis‐jasmone is related structurally to jasmonate, but exhibits a different signalling mechanism and is volatile by having lost the carboxylic acid group rather than via methylation. It also exhibits potentially useful plant growth‐promoting properties.^6^ The highly volatile plant stress‐related compound indole has been shown to be an essential priming signal in maize.^33^

### 4.2 Induction by pest‐derived non‐volatile signals

In pioneering work by Tumlinson *et al*., volicitin, an amino acid conjugate of an oxidised fatty acid, was identified from regurgitant from a caterpillar, *Spodoptera exigua*, Noctuidae, that, when applied via a wound to the plant, was an elicitor of maize volatiles attracting parasitoids.^34^ However, so far practical developments have been difficult because of the need for initial plant wounding.

In the push‐pull system for small‐holder cereal farming, companion plants are used to release the stress‐related defence signals DMNT and TMTT. The production of these compounds can be induced in certain maize plants, for example the ancestors, the teosintes, and the landraces and open pollinated varieties (OPVs) derived from these, by oviposition by the stem borer moths, *C. partellus*.^35^, ^36^, ^37^ Hybrid maize does not normally contain such a trait unless from a breeding programme without use of insecticides. Results from a genome‐wide association study (GWAS) for the associated genetics from a wide range of cultivars and OPVs have been obtained and a publication is in preparation. In the meantime the egg‐related elicitor is being studied. This elicitor has the advantage over known larval‐derived elicitors in that although the effect is systemic the elicitor is perceived by plants only bearing eggs and without damage. This has then been confirmed by similar induction using only an ethanolic solution of the lipoidal material found beneath the egg mass.^35^ Analysis by bioassay guided fractionation has allowed identification of the active components and a publication is in preparation (Midega, CAO *et al*. under preparation). The synthetic elicitor is being used by the authors in further development of this valuable trait to produce more effective companion plants and in GM‐based strategies.

## 5 SUMMARY

The evidenced and further potential value of exploiting secondary metabolites for removing constraints to sustainable food production has been discussed. Many still believe that we do not need to embrace such radical changes to current agricultural practice. However, the fact that agriculture is not going to become simpler suggests the need to accept knowledge‐intensive solutions that are sustainable. This inevitably threatens those current inputs that are energy intensive. For sustainable intensification of small‐holder farming we have companion planting, particularly the push‐pull system, which by dealing with current constraints can raise cereal production threefold with no seasonal inputs whilst at the same time potentiating animal husbandry and a range of other advantages. Controlling insects and weeds by breeding is a remote possibility without GM and its associated new developments but will remain inappropriate unless provided to the vast majority of resource‐poor small‐holder farmers via OPVs for on‐farm seed saving, for which we, as yet, have no business plan. A business plan for GM, including delivery of perennial planting material and seed for saving by farmers, can deliver, but it must be developed much faster before food shortages cause more social problems. In such developments we must not forget the small‐holder farmers who have nowhere else to go and must be helped to attain dramatically higher yields with completely sustainable technologies like push‐pull. The acceptance of many of these technologies will be promoted essentially by what agro‐ecological innovation or GM can offer in relation to the need for radically increased sustainability and intensity of food production.
